# Video Laryngoscope Assistance in Button Battery Retrieval

**DOI:** 10.1155/2023/8550685

**Published:** 2023-09-06

**Authors:** Sandy Ren, Heitor Lopes, Neil Masters

**Affiliations:** ^1^Department of Anesthesiology, Perioperative Medicine and Pain Management, Miller School of Medicine, University of Miami, Miami, Florida, USA; ^2^Department of Anesthesiology, Perioperative Medicine and Pain Management, Jackson Memorial Hospital, Miami, Florida, USA

## Abstract

Foreign body ingestion remains a common cause of pediatric emergency surgery with button battery ingestion of particular concern. Newer, higher power lithium batteries can cause catastrophic damage of the gastrointestinal tract through erosion of mucosa into surrounding structures. Prompt diagnosis and treatment are paramount. We present a case of an 11-month-old with a button battery lodged in the proximal esophagus. The extraction was difficult and only made possible with the assistance of a video laryngoscope. We make the case for more routine usage of video laryngoscopy for removal of foreign bodies in the upper esophagus.

## 1. Introduction

Young children often explore the world by placing objects in their mouths. As a result, a common chief complaint among pediatric emergency department visits is foreign body ingestion. A retrospective analysis reviewing data from 1995 to 2015 estimated over 700,000 incidents of foreign body ingestion by children less than 6 years old with objects such as coins, toys, jewelry, and batteries [1]. Button battery ingestion is of particular concern in recent years due to the increasing number of devices requiring more powerful lithium batteries with larger energy capacitances and an incidence of ingestion of over 3,000 cases reported each year in the United States [2, 3].

Batteries can cause localized tissue damage wherever they are lodged but are especially dangerous when swallowed as the battery can generate an electrical current when the positive and negative poles of the battery contact mucosal fluid [4]. Subsequent electrolysis generates hydroxide, leading to liquefactive necrosis in as little as fifteen minutes after contact with the mucosal environment [3]. Within two hours, tissue damage can become clinically significant with the feared injury of life-threatening perforation and fistula formation after twelve hours. Prompt recognition and removal are essential in any case of battery ingestion. We present the case of an 11-month-old girl who swallowed a button battery and the events that followed.

## 2. Case Description

An 11-month-old girl presented to a community emergency department after suspected foreign body ingestion and two episodes of emesis. Radiographs from the outside hospital (and later confirmed with our own fluoroscopic images) revealed an ingested button battery in the proximal esophagus at the level of the thoracic inlet. The patient was subsequently transferred to our center where pediatric surgery and anesthesiology were promptly consulted. On examination, the patient was awake, alert, intermittently crying, and maintaining her own airway.

The patient was brought to the operating room approximately seven hours after battery ingestion. Standard ASA monitors were applied, and rapid sequence induction was performed to secure the airway. The patient was intubated using a size 2 Glidescope video laryngoscope, and general anesthesia was maintained using inhaled sevoflurane. Given the proximal location of the battery on imaging, Magill forceps were immediately available should the battery be visible during video laryngoscopy, but the battery was not visualized during intubation.

The pediatric surgical team attempted multiple times to grasp the battery with different endoscopes and graspers; however, each attempt failed to retrieve the battery from the proximal esophagus. The battery appeared degraded, and standard grasping techniques were not sufficient to pull the battery past the proximal esophageal sphincter where it appeared to be lodged. A combined approach was suggested where the anesthesiology team would use the video laryngoscope to specifically visualize the posterior oropharynx and increase exposure in conjunction with further attempts by the surgical team. On the video laryngoscope screen, a small portion of the battery was clearly visible and lodged longitudinally at the esophageal inlet. One further attempt by the surgical team using the endoscope and the grasper was attempted, and again the grasper could not supply sufficient grip to remove the degraded battery. A subsequent attempt using the Magill forceps and just the video laryngoscope was immediately successful in removing the battery.

The patient tolerated the procedure with no acute complications and was extubated prior to transfer to the pediatric intensive care unit for observation following battery removal where she received broad-spectrum antibiotics. Follow-up imaging confirmed removal of the battery, and the patient tolerated an oral diet on postoperative day one with no issues. She was discharged on postoperative day two after an uneventful postoperative course.

## 3. Discussion

The use of newer, higher power, larger diameter button batteries in the era of modern electronics has led to an approximate seven-fold increase in morbidity and mortality from button battery ingestion placing greater emphasis on prompt diagnosis and treatment [5]. Mobilization of a team trained at performing pediatric endoscopy or esophagoscopy is key to prevent potential catastrophic outcomes such as esophageal perforation, aorto-esophageal fistula formation, trachea-esophageal fistula formation, and recurrent laryngeal nerve injury [2]. In this case, our patient was transferred from an outside institution many hours after battery ingestion. Our emergency team was rapidly mobilized, but attempts to remove the battery proved challenging, further prolonging the time the battery was in contact with esophageal mucosa. Only when we switched to the less conventional technique of using video laryngoscopy were we finally successful in removing the battery.

**Figure 1:**
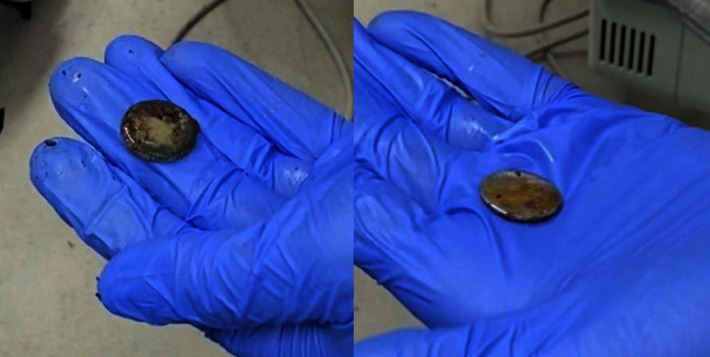
Corrosion on both sides of button battery after removal from the esophagus.

When examining the literature, case reports and observational studies of similar clinical scenarios were found in which laryngoscopy (direct or video) was a key component for foreign body removal in conjunction with a variety of instruments such as Magill forceps, hemostats, or alligator forceps [5–10]. Specifically, Kaufman et al. demonstrated that a Miller 3 video laryngoscope was successful in recovering foreign bodies from the proximal esophagus in all 22 patients in their observational study. In this study, the primary method of retrieval was the video laryngoscope which differed from our scenario where the surgical team struggled retrieving the battery. In our case, the patient's anatomy and degree of degradation of the battery prevented its retrieval with traditional surgical techniques. Video laryngoscopy became instrumental in allowing a coordinated approach to remove the battery from the esophageal inlet. We believe that this echoes the findings of Kaufman et al. and that if not used as a primary method of retrieval, video laryngoscopy should be considered as an adjunct to any attempts being made by the surgical team. Specifically, the addition of video laryngoscopy increased exposure of the posterior oropharynx and provided an alternative view to all providers in the room. This allowed the anesthesiologist and surgeon to simultaneously assess how the traditional endoscopic grasper was failing and provided a direct view of a portion of the battery such that the larger Magill forceps easily grasped and retrieved the battery. Had we attempted this technique sooner, the battery would have been removed in a timelier fashion, preventing the degree of caustic degradation of the hydrolyzed battery (Figure 1).

Furthermore, we believe that video laryngoscopy, when available, should be the de facto means for intubation in patients with proximal esophageal foreign bodies as demonstrated by Kaufman et al. If any portion of the foreign body was visible during intubation (as was later the case), it can be extracted quickly and easily using Magill forceps or similar grasping devices consistent with previous case reports and studies. In the case of button batteries, this is especially important as time is of the essence. If not visible on intubation, then video laryngoscopy should be considered as an adjunct in any situation where there is difficulty removing a foreign body from the proximal esophagus as we described above. Given the potential catastrophic nature of button battery ingestion, we echo Kaufman et al. in suggesting that any institution with a pediatric anesthetist and the resources to intubate children should attempt to expediently remove the battery prior to attempts to transfer the patient to a more specialized institution [10].

Fortunately, the patient recovered from button battery ingestion and retrieval with no complications in the perioperative and postoperative course despite the prolonged duration of ingestion. Had the patient been older than one year of age, her care could have been improved by implementing the National Capital Poison Center's guideline of preoperative administration of honey or sucralfate—a protective, pH-neutralizing, viscous barrier—as it took several hours for the patient to be transferred to a center capable of removing the foreign body. [4, 11–13]. A solution of 0.25% acetic acid could also have been utilized as an irrigating solution to neutralize the tissue after confirming there was no esophageal perforation [14, 15]. The possibility of serial MRIs should have been discussed to surveil potential subsequent tissue injury after battery removal as recommended by the North American Society of Pediatric Gastroenterology, Hepatology, and Nutrition [16, 17]. In addition, as a core message of this case, we should have transitioned to video laryngoscopy assisted retrieval earlier after the battery was found to be positioned at the esophageal inlet and traditional techniques continued to fail.

In conclusion, button battery ingestion is a time sensitive scenario requiring prompt action. Our case of an 11-month-old patient emphasizes the importance of using video laryngoscopy as an additional tool for foreign body retrieval in the proximal esophagus. This technique provides further information for both surgical and anesthesiology teams and can help expedite the removal of foreign bodies such as button batteries when other techniques fail or be a primary method of removal as has been reported in previous case reports and studies.

## Abbreviations

ASA: American Society of Anesthesiologists.

## References

[1] Orsagh-Yentis D., McAdams R. J., Roberts K. J., McKenzie L. B. (2019). Foreign-body ingestions of young children treated in US emergency departments: 1995-2015. *Pediatrics*.

[2] Eliason M. J., Ricca R. L., Gallagher T. Q. (2017). Button battery ingestion in children. *Current Opinion in Otolaryngology & Head and Neck Surgery*.

[3] Eck J. B., Ames W. A. (2020). Anesthetic implications of button battery ingestion in children. *Anesthesiology*.

[4] Hoagland M. A., Ing R. J., Jatana K. R., Jacobs I. N., Chatterjee D. (2020). Anesthetic implications of the new guidelines for button battery ingestion in children. *Anesthesia & Analgesia*.

[5] Leinwand K., Brumbaugh D. E., Kramer R. E. (2016). Button battery ingestion in children: a paradigm for management of severe pediatric foreign body ingestions. *Gastrointestinal Endoscopy Clinics of North America*.

[6] Shibuya S., Azuma T., Lane G. J., Okawada M., Yamataka A. (2020). Successful strategy for the conservative management of acquired tracheoesophageal fistula due to lithium button battery ingestion. *European Journal of Pediatric Surgery Reports*.

[7] Duan Q., Zhang F., Wang G. (2021). Vocal cord paralysis following lithium button battery ingestion in children. *European Journal of Pediatrics*.

[8] Bettadahalli V., Kumar S., Shukla I., Nair R., Kumar P. (2021). Evolving trends of button battery ingestion in Indian children at a tertiary care hospital. *Pediatric Emergency Care*.

[9] Khorana J., Tantivit Y., Phiuphong C., Pattapong S., Siripan S. (2019). Foreign body ingestion in pediatrics: distribution, management and complications. *Medicina*.

[10] Kaufmann J., Grozeva B., Laschat M. (2021). Rapid and safe removal of foreign bodies in the upper esophagus in children using an optimized Miller size 3 video laryngoscope blade. *Pediatric Anesthesia*.

[11] Lerner D. G., Brumbaugh D., Lightdale J. R., Jatana K. R., Jacobs I. N., Mamula P. (2020). Mitigating risks of swallowed button batteries: new strategies before and after removal. *Journal of Pediatric Gastroenterology and Nutrition*.

[12] Soto P. H., Reid N. E., Litovitz T. L. (2019). Time to perforation for button batteries lodged in the esophagus. *The American Journal of Emergency Medicine*.

[13] Anfang R. R., Jatana K. R., Linn R. L., Rhoades K., Fry J., Jacobs I. N. (2019). pH-neutralizing esophageal irrigations as a novel mitigation strategy for button battery injury. *The Laryngoscope*.

[14] Sethia R., Gibbs H., Jacobs I. N., Reilly J. S., Rhoades K., Jatana K. R. (2021). Current management of button battery injuries. *Laryngoscope Investigative Otolaryngology*.

[15] Jatana K. R., Barron C. L., Jacobs I. N. (2019). Initial clinical application of tissue pH neutralization after esophageal button battery removal in children. *The Laryngoscope*.

[16] Riedesel E. L., Richer E. J., Sinclair E. M. (2020). Serial MRI findings after endoscopic removal of button battery from the esophagus. *American Journal of Roentgenology*.

[17] Sinclair E. M., Stevens J. P., McElhanon B. (2021). Development and repair of aorto-esophageal fistula following esophageal button battery impaction: a case report. *Journal of Pediatric Surgery Case Reports*.

